# Understanding the Lived Experience of Family Caregivers of Loved Ones in Long-Term Care During COVID-19 Through Art

**DOI:** 10.3390/ijerph23010131

**Published:** 2026-01-21

**Authors:** Tracy M. Christianson, Evans Appiah-Kusi, Jordan Bremner

**Affiliations:** 1 School of Nursing, Thompson Rivers University, 805 TRU Way, NPH 256, Kamloops, BC V2C 0C8, Canada; appiahkusi_evans@yahoo.com; 2 Faculty of Arts, Thompson Rivers University, 805 TRU Way, NPH 256, Kamloops, BC V2C 0C8, Canada; bremnerj15@mytru.ca

**Keywords:** family caregivers, long-term care, COVID-19, visitation restrictions, arts-based research, nursing, policies

## Abstract

**Highlights:**

**Public health relevance—How does this work relate to a public health issue?**

- This study highlights how COVID-19 visitation restrictions, implemented as public health measures, unintentionally caused harm to residents and family caregivers, revealing structural weaknesses in long-term care (LTC) systems.
- Lockdowns and exclusion policies led to trauma, grief, and burnout among caregivers, making this a significant public health concern related to mental health and social determinants of health.

**Public health significance—Why is this work of significance to public health?**

- The findings show that policies designed to protect physical health can negatively affect emotional and psychological health, emphasizing the need for holistic approaches.
- Recognizing family caregivers as integral to care delivery is crucial for improving health outcomes and reducing systemic strain during crises.

**Public health implications—What are the key implications or messages for practitioners, policymakers, and/or researchers in public health?**

- Public health policies should embed family involvement and trauma-informed practices in LTC, even during emergencies, to prevent isolation-related harm.
- Practitioners and policymakers must prioritize caregiver mental health resources, culturally safe care models, and flexible visitation protocols to mitigate future crises.

**Abstract:**

Public health restrictions during COVID-19 disproportionately affected older adults, especially those in long-term care (LTC) and their families. Family caregivers (FCs) are essential care partners in LTC settings, yet pandemic policies largely excluded them, creating emotional and systemic consequences. This study explored FCs’ experiences of visitation restrictions in British Columbia, Canada, using an arts-based qualitative approach within a larger mixed-methods project. Eight FCs participated in completing a total of twelve artworks, including photographs, collages, and creative writing that reflected their experiences. Virtual talking circles were used to facilitate the sharing and description of their experiences. Findings revealed three interconnected theme categories with eleven sub-themes. These themes suggest a plausible pathway: infection-control rules may lead to caregiver exclusion, disrupting relational continuity and oversight and contributing to distress and task-centered care. While context-specific and exploratory, results underscore the need for trauma-informed, family-inclusive policies and cultural safety in LTC. Arts-based research methods provided a powerful lens for capturing emotional realities often missed by conventional research.

## 1. Introduction

Family caregiving is essential in long-term care (LTC), as it provides personalized attention often missing in institutional settings, especially for older adults and those with chronic illnesses [1]. Family caregivers (FCs) offer around 25 h of care weekly, providing emotional support, daily assistance, and advocacy [2,3]. Despite their crucial role, FCs face significant emotional, physical, and financial challenges, including high stress, burnout, and adverse health outcomes [2,3]. In this report, the authors used an arts-based research approach to explore FCs’ experiences and the impact that visitation restrictions had on them during the COVID-19 pandemic. Arts-based research is a novel method to explore individuals’ experiences related to complex and emotionally charged phenomena. This approach provides deeper insight into personal and relational dimensions that traditional qualitative methods may overlook.

### 1.1. The Impact of COVID-19 on Caregivers and Their Loved Ones

The COVID-19 pandemic exacerbated FCs’ struggles in LTC. Lockdowns and visitation restrictions increased distress, anxiety, and feelings of powerlessness [4]. Disruptions in caregiving routines affected FCs’ mental health due to uncertainty about their loved ones’ well-being and their inability to fulfill caregiving roles [5,6]. The pandemic also exposed structural inadequacies in LTC settings, complicating caregiving during the crisis [7].

### 1.2. Exploring Lived Experiences Through Art

In 2022, the researchers conducted a larger study exploring the lived experiences of family caregivers in LTC settings in British Columbia, Canada, during the COVID-19 pandemic. The third phase of the study utilized art as a medium for self-expression to understand the impact COVID-19 restrictions had on FCs more deeply. Art as a research tool offers a novel and effective means of exploring and expressing complex lived experiences, particularly in the context of caregiving. Although arts-based research provides an avenue for participants to convey emotions, thoughts, and experiences that may be difficult to articulate through traditional methods [8], allowing for rich, multilayered interpretations of participants’ experiences and facilitating a deeper understanding of their psychological and emotional states [9], its use in this study context was limited. Using art to explore caregivers’ experiences provides a means to comprehensively capture their emotional states, offering a more detailed and empathetic view of the challenges they faced [10].

## 2. Materials and Methods

As part of a larger mixed-methods study that received harmonized ethical approval (H21-01256) from both Thompson Rivers University’s and the University of British Columbia–Okanagan’s Ethical Review Boards, FCs over the age of 19 years with a loved one living in LTC were recruited through social media platforms, newspapers, and community posters. Participants who completed the study’s online survey or one-on-one interviews were recruited for the arts-based talking circles. The FCs (spouses or adult children) were mailed art supplies such as writing pads, paints, and other craft materials and a guide asking them to express their feelings and experiences of being restricted from their loved ones in LTC due to COVID-19 visitation policies. The guide provided an example of art and encouraged participants to express their feelings using whatever medium they felt comfortable with, even beyond the provided supplies, such as photography. Due to the pandemic and the need for social distancing, participants created their art before the virtual talking circles, which were hosted on Microsoft Teams. 

Rather than serving merely as visual sources of data, the artworks served primarily as a means to evoke participants’ feelings and experiences and to facilitate the conversations during the talking circles, grounding the interpretation of the artworks in participants’ narrated meaning. An icebreaker activity helped participants feel comfortable before they described their art. Each talking circle was facilitated by Research Assistants, EAK and JB, with the lead researcher, TC present. Participants were given ample time to describe their art pieces. Participants shared only their first names to ensure confidentiality. Each session was audio recorded with participants’ consent, and two researchers took notes to capture all the discussion. The recorded transcriptions were anonymized to ensure confidentiality. Participants were encouraged to ask questions and offer thoughts, creating an engaging dialog for deeper exploration of their experiences. After the talking circles, the researchers debriefed the experience to minimize any potential biases. 

Braun and Clarke’s classical approach to thematic analysis was used [11]. Three researchers immersed themselves in the data, each reviewing and developing sets of codes from the transcripts to generate themes. The research team reviewed and discussed the data to reach consensus on the final themes using constant comparison and triangulation, ensuring the reliability and validity of the data. 

## 3. Results

Eight family members, all Caucasian, from all five health authorities in British Columbia participated in this study, comprising one male spouse (P13), one female spouse (P07), and six female adult children (P01, P03, P06, P15, P18, P20). Four participants joined one of two talking circles, where they described the art that captured their unique emotions and experiences during the COVID-19 visitation restrictions.

### 3.1. Art Creations

**Figure 1:**
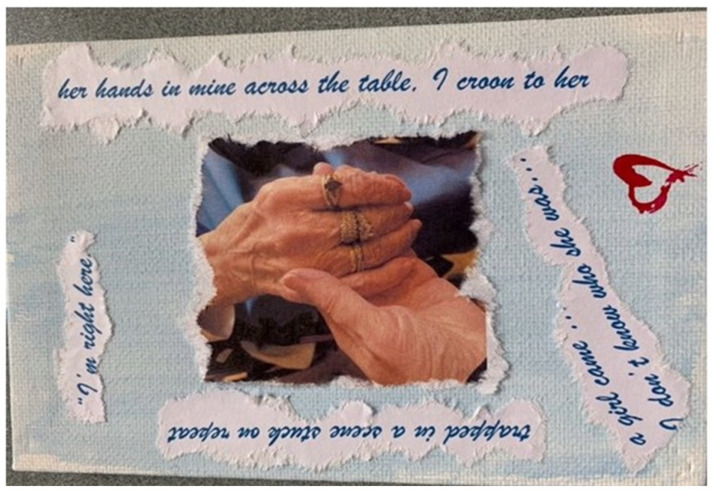
Participant 01’s art creation.

**Figure 2:**
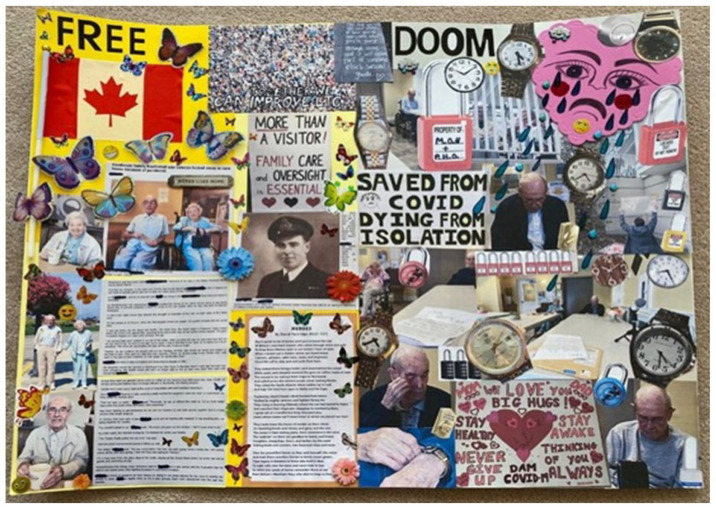
Participant 06’s art creation.

**Figure 3:**
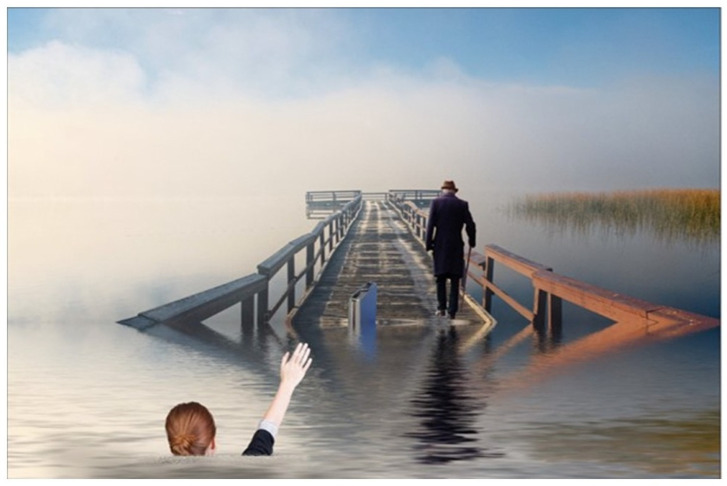
Participant 13’s art creation.

Twelve evocative art pieces, including collages, photographs, and creative writing, were created by participants to express the emotional depth of their experiences during the COVID-19 pandemic. These works served as powerful vehicles for personal storytelling and reflection. For example, Participant 01 shared, “Poetry is where I go… they say poetry can say the unsayable.” Her piece featured a photograph of her mother’s hand in hers, surrounded by fragments of a poem she wrote, symbolizing the emotional fragmentation and memory loss experienced during lockdown (see Figure 1). Participant 06’s work was a bold call to action, stating, “Speaking up and speaking out is an uncomfortable position, but some of us have to make it our mission.” Their poster was divided into two contrasting themes of “freedom” and “doom” using imagery such as butterflies and locks to illustrate the paradox of protecting LTC residents from COVID-19 while simultaneously exposing them to the harms of isolation (see Figure 2). Similarly, Participant 13 described visitation restrictions as “savage” and “cruel.” Their image depicted a person walking away from a sinking pier, with a woman in the water reaching out, symbolizing the helplessness of a vulnerable loved one and the forced emotional and physical distancing experienced by family caregivers (see Figure 3).

### 3.2. Talking Circles

Through the talking circles, participants shared their art and what it represented for them. From the two talking circles, the data revealed three theme categories with eleven sub-themes. Two categories, trauma and emotional toll, had four sub-themes each, and the third, othering, had three sub-themes. Rather than presenting themes as descriptive categories, this section interprets how participants’ narratives and art illuminate mechanisms such as fractured relational continuity, procedural disempowerment, and loss of person-centered care that shaped their lived experiences. After themes were generated, the researchers input the themes into Microsoft 365 Co-pilot, a generative artificial intelligence software, to format a table to provide a clear and effective presentation of the themes and sub-themes (see Table 1). All content was reviewed, edited, and remains the responsibility of the authors.

### 3.3. Trauma Experienced

The trauma experienced by family caregivers (FCs) was multifaceted and deeply personal. The most pervasive form was separation trauma, where lockdowns abruptly severed physical, emotional, and social connections. Participants described feeling “torn apart,” with loved ones expressing confusion and abandonment, such as, “Why have you left me?” (P13), and “Did you think I didn’t love you?” (P20). These moments seemed to create long-term psychological scars as FCs were unable to provide comfort during their loved ones’ most vulnerable times. Artworks depicting a woman reaching from sinking piers visualized this asymmetry of agency, where FCs were constrained by policy, and residents were submerged in vulnerability.

Medical neglect emerged as another traumatic dimension with FCs recounting lapses in care such as broken hearing aids, missing eyeglasses, poor hygiene, and even opioid overdoses; as P01 described, “She had opiates in her urine… they had to give her Narcan”. These experiences suggest that exclusion removed a critical layer of informal monitoring, amplifying risks of missed care. Institutionalization trauma reflected procedural harm through abrupt transitions without FCs’ consultation, for example, “She was placed under the Mental Health Act… no consultation whatsoever” (P13), reinforcing feelings of helplessness and mistrust.

Finally, pandemic-induced decline highlighted isolation-linked deterioration, as FCs observed rapid physical and cognitive deterioration in their loved ones, attributing it directly to isolation, as one participant stated, “I feel like my mom fell because of the pandemic” (P03), which left FCs to grieve not only the loss of time but also the loss of vitality and connection. 

Collectively, these sub-themes show that safety policies, when decoupled from relational care, reorganized caregiving in ways that produced enduring psychological and physical consequences. Trauma was not limited to emotional pain; it emerged as a relational and institutional phenomenon, underscoring the need for approaches that balance infection control with relational continuity.

### 3.4. Emotional Toll 

The emotional toll on FCs was profound and structurally produced, extending beyond sadness to encompass ambiguous grief, invisible labor, and moral distress. Grief and loss were not confined to death but extended to the gradual erosion of identity, connection, and shared life. “The grieving goes on for a long time” (P13) and “She just sits and exists” (P18) reflect the sorrow of watching loved ones fade in isolation. Burnout and exhaustion intensified when FCs previously providing 24/7 support were abruptly removed from caregiving roles, which left them feeling disoriented and depleted, as (P18) noted, “I cared for my mom 24/7 for 3.5 years… then I wasn’t allowed to see her again”. 

Another area of emotional toll was helplessness and anguish, which were intensified by rigid policies and a lack of responsiveness, as illustrated by, “My mom died of COVID… because the restrictions held the only human beings on Earth that could keep her lucid” (P15), which illustrates the devastating consequences of exclusion. Family caregivers were often left to witness suffering from a distance, powerless to intervene. Finally, advocacy fatigue emerged as FCs repeatedly fought for basic care and dignity. “I had to advocate … from the get-go” (P07) and “I would shout it from the mountain tops every day if I could” (P18) reveal the emotional cost of persistent advocacy in a system that often resisted change. Such experiences were not isolated reactions but rather consequences of policies that reframed essential caregiving as visitation, stripping caregivers of their agency and deepening moral distress.

### 3.5. Sense of Othering

The theme of othering refers to the systemic marginalization of residents and their FCs, where care practices and policies positioned them as outsiders rather than integral partners. Othering manifested through three interrelated sub-themes: dehumanization of residents, family exclusion, and cultural disconnection.

Dehumanization occurred when residents were reduced to tasks rather than being seen as individuals with dignity, as exemplified by “They treat them as a generic bunch of I don’t know what” (P01), and “Is it really care if it is not caring?” (P03). Family exclusion was evident by gatekeeping practices that denied access, voice, and agency. “There was no consultation whatsoever” (P13), and “They told me they were going to put a restraining order against me” (P06) were examples of this exclusion. These actions not only isolated FCs but also undermined their essential caregiving roles. 

Othering was also expressed as cultural disconnection, adding another layer of alienation. Participants critiqued, “I see long-term care as being a product of colonization” (P03), highlighting care models that fail to reflect diverse cultural values and relational caregiving practices and traditions of families. Concerns about the invisibility of aging populations were also shared, as clearly expressed by Participant 18, “We are headed for much bigger problems… aging is easy to ignore”, reinforcing this sense of systemic inequities and marginalization. Across all themes, the data illustrate a clear policy-to-practice trajectory where infection-control policies excluded family caregivers that disrupted relational continuity and oversight. This exclusion heightened risk and functional decline in loved ones’ health and caused moral distress for FCs, resulting in a system that normalized a sense of othering. Despite these challenges, FCs demonstrated resilience, finding solace in small moments of connection, spiritual strength, and collective advocacy. The talking circles provided a space for shared grief, validation, and hope, reinforcing the importance of relational support in healing trauma. 

## 4. Discussion

This study offers an exploratory understanding of the lived experiences of FCs across British Columbia, Canada, during COVID-19 LTC visitation restrictions, using an arts-based methodology. While the findings are important, they should be interpreted with caution, given the small, purposive sample and context-specific setting. The researchers’ interpretations aim to illuminate plausible mechanisms, such as disrupted relational continuity, procedural disempowerment, and erosion of person-centered care, that seem to have shaped participants’ experiences. When we connect these findings to the prior literature, we do so to situate the results and contribute to the literature on the importance of the role of FCs in LTC. 

### 4.1. Trauma, Emotional Toll and the Invisible Labor of Caregiving

Participants’ narratives and artworks suggest that trauma was experienced not only as emotional distress but also as a relational and systemic phenomenon. Forced separation disrupted the caregiving ties that help maintain residents’ orientation, dignity, and daily functioning. The FCs’ report of missed care and reduced oversight may reflect the removal of informal monitoring typically provided by families, which could heighten the risk of undetected lapses. Decisions made without family input, such as abrupt transitions or restrictive gatekeeping, were described as procedurally harmful, irrespective of clinical intent. These observations are consistent with the literature that emphasizes relational safety and trauma-informed approaches in LTC, but in this study they remain contextual and interpretive rather than generalizable findings [4,12]. Accordingly, the results suggest that COVID-19 restriction policies may benefit from approaches that explicitly consider relational continuity alongside physical safety.

The emotional burden described by FCs appeared to stem from constrained caregiving roles and persistent advocacy for basic care. The FCs who had provided intensive, hands-on support prior to the pandemic reported that their enforced absence led to ambiguity, cumulative grief, and a sense of moral distress when they could not act according to their values. While these accounts align with studies documenting psychological strain among FCs during COVID-19 [4,13,14], the qualitative data in this study cannot establish magnitude or prevalence. Therefore, these insights are framed as plausible patterns warranting further examination with larger and more diverse samples. Future work should evaluate whether flexible, family-inclusive practices can mitigate moral distress and sustain caregiving roles without compromising infection control as others have also suggested [15,16,17,18].

### 4.2. Othering and the Need for Cultural and Structural Reform

The pandemic highlighted systemic biases favoring facility-centered over family-centered caregiving [4,12,13,17,18]. Visitation restrictions isolated FCs and limited their access to loved ones as other studies have found. For example, Christianson et al. found that FCs were excluded despite their essential caregiving roles, intensifying their sense of othering [5]. Similarly, bureaucratic barriers and limited communication tended to further FCs’ sense of helplessness and alienation, undervaluing FCs’ contributions and deepening their vulnerability [13,17,18,19].

The sense of othering experienced by FCs revealed care environments where taskification appeared to displace personhood and where gatekeeping reclassified FCs from partners to outsiders. The dehumanization of residents and exclusion of families as caregivers point to a care model with structural and cultural tensions between efficiency and empathy as well as care routines and family partnership that merit further study. Cultural disconnection further alienated those whose caregiving values are rooted in relational and community-based traditions [20]. The recurrent imagery of barriers and fragmentation in participants’ art can be perceived as being symbolic of exclusion; however, these findings were gathered using an interpretive lens rather than a definitive system diagnosis. However, given the findings of other studies on this phenomenon, the current findings lean toward a systemic issue [13,17,18,19].

### 4.3. Art in Research

Arts-based research allowed FCs to create art as a means to express and share their experiences of LTC visitation restrictions, capturing the complexities of their experiences more deeply. In this study, participants found the arts-based talking circles to be validating, empowering, and therapeutic. Additionally, participants expressed gratitude for the opportunity to share their stories and art and build solidarity with others. In the only other study found that used arts-based research with FCs, Boamah et al. found that photovoice, which involved participants taking photographs, allowed caregivers to portray their experiences, generating rich insights into emotional impacts and coping mechanisms [21]. Traditional qualitative methods offer insights, but arts-based research provides a richer understanding of the experience.

## 5. Limitations

The small, purposive sample limits the generalizability of the findings and may not represent the broader population of FCs. Additionally, the lack of racial and cultural diversity limits insights into how visitation restrictions intersected with systemic racism and culturally specific caregiving practices. Therefore, the experiences and emotional impacts described may not apply universally. However, qualitative studies focus on lived experiences to gain a more intimate understanding of the topic. To ensure trustworthiness in this qualitative phase, several strategies were employed. Braun and Clarke’s thematic analysis guided the process [10], with three researchers independently coding transcripts and using constant comparison to refine themes, enhancing credibility and dependability. Consensus meetings and triangulation comparing art pieces with participants’ narratives strengthened confirmability. Audio recordings were anonymized, and participants used first names only to maintain confidentiality. Reflexivity was supported through researcher debriefing after each session to minimize bias. Finally, rich descriptions of themes and illustrative quotes were provided to promote transferability, acknowledging limitations related to sample size while offering sufficient context to judge applicability.

### Implications for Nursing Practice and Policy

This study suggests that FCs should be recognized as essential partners in LTC. Participants’ experiences of emotional trauma and systemic exclusion highlight the need for trauma-informed, relational care models. Nurses and administrators may consider policies that support family involvement, flexible visitation, and transparent communication, especially during public health emergencies. Practical steps include family engagement protocols, staff training in empathy and cultural safety, and feedback mechanisms that incorporate FCs’ perspectives. These measures may help reduce distress and improve resident outcomes.

Findings point to a plausible pathway by which infection-control restrictions or rules may lead to caregiver exclusion, disrupting relational continuity and oversight, and ultimately contributing to emotional distress, perceived neglect, and the normalization of task-centered care. It is presented as a hypothesis-generating framework. Future research should test these dynamics with larger samples and evaluate family-inclusive practices that balance safety with relationships. Policy changes should proceed cautiously, starting with pilot interventions and monitoring for unintended consequences.

## 6. Conclusions

This study explored the lived experiences of FCs during COVID-19 LTC visitation restrictions using an arts-based approach. The findings suggest that enforced separation and institutional gatekeeping disrupted relational caregiving, contributing to emotional distress and perceived systemic gaps. While these insights are context-specific and drawn from a small sample, they underscore the importance of considering family inclusion and relational continuity in future policy and practice. Further research with larger and more diverse samples is needed to further examine these dynamics and evaluate strategies that balance infection control with emotional and relational well-being.

## Figures and Tables

**Table 1:** Talking circle themes and example quotes *.

Theme Category	Theme	Description	Example Participant Quotes
**1. Trauma Experienced**	**Separation Trauma**	Forced separation during lockdowns caused deep emotional pain.	“What she said to me before the lockdown was ‘I would go insane without your visits.’”—P13
			“Why have you left me?”—P13 stating what wife shared
			Did you think I didn’t care? Did you think I didn’t love you? Did you think I deserted you?”—P20
			It was the most difficult time not to be there and hold your hand– P20
			She was terrified, wild as recent prey… She knows she cannot find the words she needs.”—P01
	**Medical Neglect**	Lack of adequate care in hospitals and long-term care.	“For 10 days they didn’t even… they only got her out of bed once.”—P03
			“I don’t think they even washed behind his ears until one time I looked… and it was gross.”—P07
			She broke her hip… had surgery that night and was walking on it the next day—P18
			Her hearing aids were broken while she was in care.”—P20
			My mom’s hearing aids were missing… her eyeglasses went missing… I don’t know if I’m losing her smile because of dementia or medication.”—P18
			She had opiates in her urine… they had to give her Narcan in emergency—P01
	**Institutionalization Trauma**	Abrupt transitions and lack of consultation.	“She was placed under the Mental Health Act… no consultation whatsoever.”—P13
			Without informing me beforehand, she was sent back to care.”—P18
	**Pandemic-Induced Decline**	Physical deterioration is linked to isolation.	“I feel like my mom fell because of the pandemic.”—P03
			My dad became very, very despondent.”—P06
**2. Emotional Toll**	**Grief and Loss**	Ongoing grief over cognitive and physical decline.	“The grieving goes on for a long time.”—P13
			“It’s a whole big change for both of us.”—P07
			I shed many tears.”—P20
			My mom doesn’t have a dance partner anymore… she just sits and exists.”—P18
	**Burnout and Exhaustion**	Caregivers are overwhelmed by responsibilities.	“I found I was quite burnt out… I’m a university student as well.”—P03
			I cared for my mom 24/7 for 3.5 years… then I wasn’t allowed to see her again.”—P18
	**Helplessness and Anguish**	Feeling powerless against institutional rules.	“My mom died of COVID… because the restrictions held the only human beings on Earth that could keep her lucid.”—P15
			I wanted my mom out of there… I wanted her in emergency now.”—P06
			I’m already broken, I think from this experience.”—Wendy
	**Advocacy Fatigue**	Constant need to fight for basic care.	“I had to advocate for Hank… from the get-go.”—P07
			I would shout it from the mountain tops every day if I could.”—P18
**3. Sense of Othering**	**Dehumanization of Residents**	Residents treated as tasks, not people.	“Is it really care if it is not caring?”—P03
			They served those people food that was stone cold.”—Liz
			“These are people. They have dignity.”—P13
			They treat them as a generic bunch of I don’t know what.”—P01
	**Family Exclusion**	Family caregivers denied access and voice.	“There was no consultation whatsoever.”—P13
			They wouldn’t let me take him out to get his hearing aids replaced.”—P06
			“I was not allowed to come in anymore.”—P07
			I wasn’t allowed to see her again until she broke her hip.”—P18
			We were told we couldn’t be essential visitors—Multiple participants
			They told me they were going to put a restraining order against me.”—P06
	**Cultural Disconnection**	Long-term care is seen as a colonial construct.	“I see long-term care as being a product of colonization.”—P03
			We are headed for much bigger problems… aging is easy to ignore.”—P18

* Generative AI tool, M365 Copilot (GPT 5 Model, 2025) was used to create the table. The authors have reviewed and edited the output and take full responsibility for the content.

## Data Availability

The raw data supporting the conclusions of this article will be made available by the authors on request.
